# Exploration of *Parascaris* species in three different *Equus* populations in China

**DOI:** 10.1186/s13071-023-05768-3

**Published:** 2023-06-15

**Authors:** Mengchao Zhou, Yaxian Lu, Lei Han, Maolin Lu, Chunyu Guan, Jie Yu, Hetong Liu, Denghui Chen, Hongjia Li, Yuling Yang, Lu Zhang, Lihong Tian, Quan Liu, Zhijun Hou

**Affiliations:** 1 College of Wildlife and Protected Area, Northeast Forestry University, Harbin, China; 2 Laboratory of Vector-Borne Diseases and Pathogens Ecology, Northeast Forestry University, Harbin, China; 3 Harbin Northern Forest Zoo, Harbin, China; 4 Dong-E-E-Jiao Co. Ltd, Shandong, China

**Keywords:** *Equus*, Karyotype, Phylogenetic analysis, *Parascaris* spp.

## Abstract

**Background:**

The roundworms, *Parascaris* spp., are important nematode parasites of foals and were historically model organisms in the field of cell biology, leading to many important discoveries. According to karyotype, ascarids in *Equus* are commonly divided into *Parascaris univalens* (2*n* = 2) and *Parascaris equorum* (2*n* = 4).

**Methods:**

Here, we performed morphological identification, karyotyping and sequencing of roundworms from three different hosts (horses, zebras and donkeys). Phylogenetic analysis was performed to study the divergence of these ascarids based on cytochrome c oxidase subunit I (*COI*) and internal transcribed spacer (ITS) sequences.

**Results:**

Karyotyping, performed on eggs recovered from worms of three different *Equus* hosts in China, showed two different karyotypes (2*n* = 2 in *P. univalens* collected from horses and zebras; 2*n* = 6 in *Parascaris* sp. collected from donkeys). There are some differences in the terminal part of the spicula between *P. univalens* (concave) and *Parascaris* sp. (rounded). Additionally, it was found that the egg’s chitinous layer was significantly thicker in *Parascaris* sp. (> 5 μm) than *P. univalens* (< 5 μm) (*F*_(2537)_ = 1967, *P* < 0.01). Phylogenetic trees showed that the sequences of *Parascaris* from *Equus* hosts were divided into two distinct lineages based on sequences of the *COI* and ITS.

**Conclusions:**

Comparing the differences in roundworms collected from three different *Equus* hosts, this study describes a *Parascaris* species (*Parascaris* sp.) with six chromosomes in donkeys. It is worth noting that the thickness of the chitinous layer in the *Parascaris* egg may serve as a diagnostic indicator to distinguish the two roundworms (*P. univalens* and *Parascaris* sp.). The *Parascaris* sp. with six chromosomes in donkeys in the present study may be a species of *P. trivalens* described in 1934, but the possibility that it is a new *Parascaris* species cannot be ruled out. Both karyotyping and molecular analysis are necessary to solve the taxonomic problems in *Parascaris* species.

**Supplementary Information:**

The online version contains supplementary material available at 10.1186/s13071-023-05768-3.

## Background

The Equidae are important reservoir hosts for various nematode parasites, some of which can cause significant morbidity or mortality if their hosts are untreated. Equine roundworms are large parasitic nematodes that predominantly infect foals and weanlings. *Parascaris* have a direct life cycle where infective eggs ingested from the environment hatch in the horse’s stomach. The larvae then penetrate the intestinal mucosa, migrating through the liver and lungs, and eventually return to the small intestine to develop into adults and reproduce [1]. Infected with large numbers of adult worms, the hosts often present with coughing, anorexia, lethal intestinal obstruction or rupture, and even death [2]. Larvae in the migrating stages can also cause hepatitis, pneumonitis and associated respiratory disorders [3].

Two species of roundworms, *Parascaris univalens* and *Parascaris equorum*, are found infecting *Equus* hosts [4–6]. These two species cannot be easily distinguished morphologically but differ concerning their karyotype. One pair of chromosomes (2*n* = 2) is found in *P. univalens* and two pairs (2*n* = 4) are found in *P. equorum* [7]. Additionally, *P. trivalens*, another rare horse roundworm with three pairs of chromosomes, was described in 1934 and 1937 [8, 9]. However, there have been no reports of this species since 1937.

In 2014, Nielsen et al. karyotyped *P. univalens* and uploaded a complete mitochondrial genome of the worm to the National Center for Biotechnology Information (NCBI) database. Until now, only Goday et al. have karyotyped *P. equorum*, collected from horses in the 1980s [7, 11, 12]. However, many studies have shown that *P. univalens* is more prevalent whereas *P. equorum* cannot be found in horses using cytological methods [1, 6, 10]. Himmelstjerna et al. (2021) concluded that most *P. equorum* registered in the NCBI database based solely on cytochrome c oxidase subunit I (*COI*) and internal transcribed spacer (ITS) sequence analysis without karyotyping were actually derived from *P. univalens* specimens [13]. For this reason, the results based only on analyzing the sequences of horse roundworms are inadequate for identifying *P. univalens* and *P. equorum* at the present time.

Although cytological analysis is a useful method for specific identification, it would be desirable to have available genomic markers for polymerase chain reaction (PCR)-based analyses of genetic variation within *Parascaris*. Combined with cytological analysis, some researchers have generated mitochondrial genome sequence data for *P. univalens* to provide a reference sequence for this parasite [3, 6]. Here, we analyzed the morphology, karyotype and genetic characteristics of *Parascaris* in three *Equus* host populations of horse (*E. caballus*), zebra (*E. zebra*) and donkey (*E. asinus*) in northern China. We present the first report on the cytological analysis of *Parascaris* populations in donkeys and show that the roundworms in donkeys were a *Parascaris* species with six chromosomes.

## Methods

### Sample collection and morphology identification

**Fig. 1:**
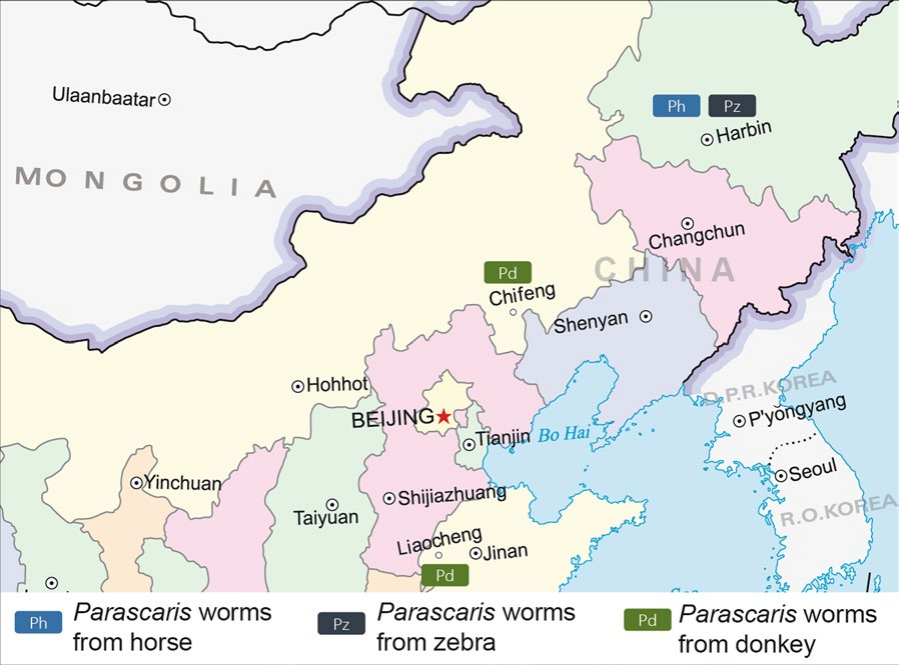
Sampling localities and geographical distribution of the three distinct populations of *Parascaris* spp.

The roundworms in the present study were collected from *Equus* hosts after anthelmintic treatment. Four horse roundworm individuals (h1–h4) were collected from a farm in Harbin, Heilongjiang, China. Twelve roundworms from zebras (z1–z12) were obtained from Harbin Northern Forest Zoo, Heilongjiang, China. Fourteen roundworms (d1–d14) from donkeys were collected from a farm in Liaocheng, Shandong, China. Another 10 roundworms from donkeys (d15–d24, karyotyping of these roundworms could not be carried out due to our poor preservation) were collected from a farm in Chifeng, Inner Mongolia, China (Fig. 1). The roundworm information in this study is presented in Additional file 2: Table S1. All 40 specimens were washed extensively in phosphate-buffered saline (PBS, 37 °C) and transported immediately to the parasitology laboratory. Here, the structure of the head, tail and spicula and the length of the body were observed with an Olympus CX43 microscope (Olympus, Tokyo, Japan) using EPview v 3.2 software (Olympus Scientific Solutions, Tokyo, Japan). Additionally, the 20 female individuals (h1–h3, z1−z8 and d1−d9) were carefully dissected, and the gonads and zygotes were collected. The size (length and width) and the chitinous layer of the eggs (20 eggs of each female individual were chosen) were measured. Then the gonads and remaining male roundworms were stored at −80 °C until further use, and zygotes were stored at 4 °C for the next karyotyping.

### Karyotyping

We performed karyotyping on the collected zygotes (20 eggs of each female individual were chosen) for analyzing early embryonic mitotic divisions as described previously [6]. Briefly, the appropriate amount of 0.5 M NaOH, 0.4 M KOH and a mixture of 6% hypochlorite and 0.4 M KOH (17:83) were added sequentially to a tube containing the eggs, and after adding each reagent, resting, resuspension, centrifugation and washing with cold distilled water were necessary for the decortication of the eggs. Then the eggs were washed and resuspended in 0.7% saline solution and incubated at 37 °C until they developed into the first or second embryonic mitotic division under the microscope. Before staining the eggs, the saltwater was sucked out, and the tubes were filled with a mixture of methanol, acetic acid and chloroform (6:3:1) and were left for 1 h. Drops of embryo suspension were deposited on slides and left at room temperature for drying. Finally, staining was carried out with 4′,6-diamidino-2-phenylindole (DAPI) for 10 min and then examined under a fluorescence microscope. Additionally, Giemsa banding staining (G-banding) was applied after the eggs were deshelled, as described previously [16].

### Nucleic acid isolation and gene amplification

DNA was extracted from all 40 *Parascaris* samples using the QIAamp DNA Mini Kit (QIAGEN, Germany) following the manufacturer’s instructions. Partial sequences of the gene of mitochondrial DNA (mtDNA), *COI* and ITS (including partial ITS1, 5.8 s and ITS2) were amplified to explore the genetic characteristics and phylogenetic relationships. Primer F5 (5′-TCATAAGGATATTGGGACC-3′) and primer F6 (5′-GCAAAATGTAAAGGGAAAA-3′) [17] were applied to amply the *COI* (996-base pair [bp]) gene of each specimen. Primers NC5 (5′-GTAGGTGAACCTGCGGAAGGATCATT-3′) and NC2 (5′-TTAGTTTCTTTTCCTCCGCT-3′) [18] were applied to amply the ITS (~770 bp) sequence of each specimen. Regarding the two PCR reactions, all the volumes were 25 μl, including 12.5 µl *Premix Taq* (*Ex Taq* version 2.0 plus dye, Takara, Japan), 8.5 µl double-distilled water ddH_2_O), 1 µl of each primer and 2 µl of template DNA under the following conditions: initial denaturation at 94 °C for 5 min, then 35 cycles at 94 °C for 30 s (denaturation); annealing at 50 (*COI*)/55 °C (ITS) for 30 s, extension at 72 °C for 90 s (*COI*)/60 s (ITS), followed by a final extension at 72 °C for 7 min. The PCR product was examined on a 1.5% agarose gel to verify that the reactions produced single bands, and then was sent to Comate Biosciences Co., Ltd. (Changchun, China) for Sanger sequencing in the forward and reverse directions.

### Phylogenetic relationship and genetic structure

Multiple sequence alignments of nucleotide sequences in this study and sequences available from GenBank were generated using ClustalX v2.0 software. DnaSP v5.10 software was applied to establish the sequence haplotypes of different populations. For *COI*, the sequences of *P. univalens* samples in horses and zebras were divided into three haplotypes (CHU1–CHU3); the *Parascaris* sp. in donkeys in Liaocheng were divided into five haplotypes (CHS1–CHS5); the sequences of samples in donkeys in Chifeng were divided into three haplotypes (CH1–CH3). For ITS, the sequences of *P. univalens* were divided into two haplotypes (IHU1 and IHU2); the *Parascaris* sp. were divided into one haplotype (IHS1); the sequences of samples in donkeys in Chifeng were divided into two haplotypes (IH1 and IH2) (Additional file 3: Table S2). For phylogenetic analysis, the representative sequences for each haplotype defined in the present study were used to analyze phylogenetic relationships. The *Ascaris suum COI* (KY045804) and ITS (KY964445) sequences were retrieved from GenBank and used as outgroups to perform phylogenetic analysis, which was hypothesized using maximum likelihood (ML) and Bayesian inference (BI). The best-fitting nucleotide substitution model was selected using Modeltest 3.7 software with the Akaike information criterion (AIC) [19]. For ML, the best models of *COI* and ITS sequences were HKY+G and T92, respectively. In addition, phylogenetic analysis was conducted using PhyML 3.0 software [20]. Bootstrap branch support values (MLBS) were obtained with 1000 rapid bootstrap inferences, and thereafter searched through ML search on the dataset. For BI, the best models of *COI* and ITS sequences were GTR+G and HKY, respectively. Phylogenetic analysis was performed using MrBayes v3.2 software [21]. The parameters were set as follows: nst = 6 (*COI* sequences)/2 (ITS sequences), rates = propinv (*COI* sequences)/equal (ITS sequences), with four Markov chain Monte Carlo (MCMC) run for two runs from random starting trees for five million generations, and the trees were sampled every 1000 generations. In addition, 25% of generations were discarded as “burn-in,” and the remaining samples were used to calculate Bayesian posterior probabilities (BPP). Phylograms were plotted using FigTree v1.4.2 software.

### Statistical analysis

To investigate the differences between the structural morphology (the size and thickness of the chitinous layer) of the *Parascaris* eggs from different hosts, the data were first tested for normality using the W-test. Then one-way analysis of variance (ANOVA) in R (v4.0.2) was used to analyze the divergence of the data. The "ggpair" function in R was used to perform correlation analysis between the size and the thickness of the chitinous layer of the eggs. The final result when using analytical signals was the complex Pearson correlation coefficient. The *P*-value was used to show the significance of the difference. Additionally, the “dplyr” package, “patchwork” package and “ggplot2” package in R were used to visualize the results.

## Results

**Fig. 2:**
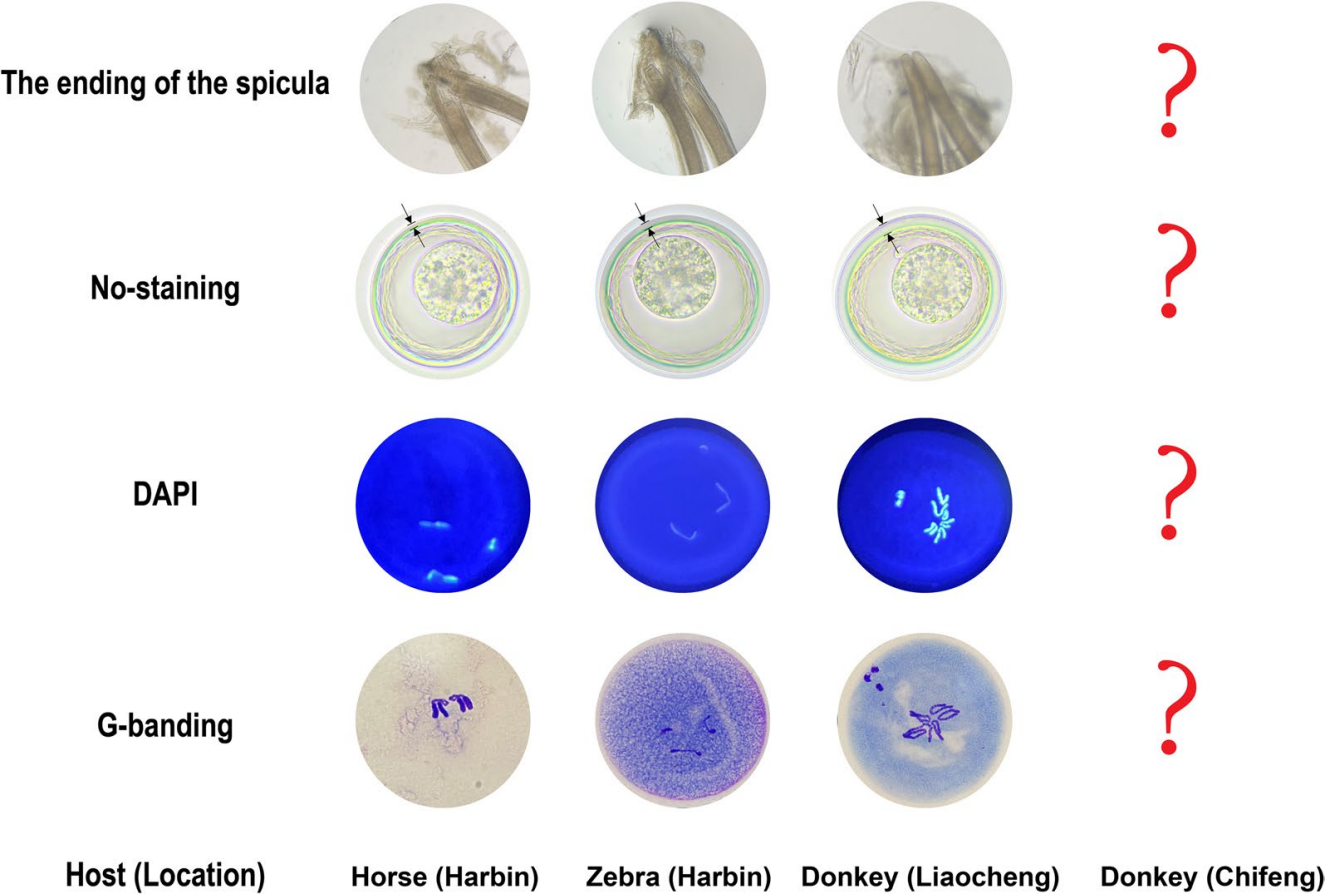
The differences in the terminal part of the spicula, the thickness of the chitinous layer and the karyotypes of *Parascaris* species from different hosts or locations. The area indicated by the arrow represents the thickness of the chitinous layer

**Fig. 3:**
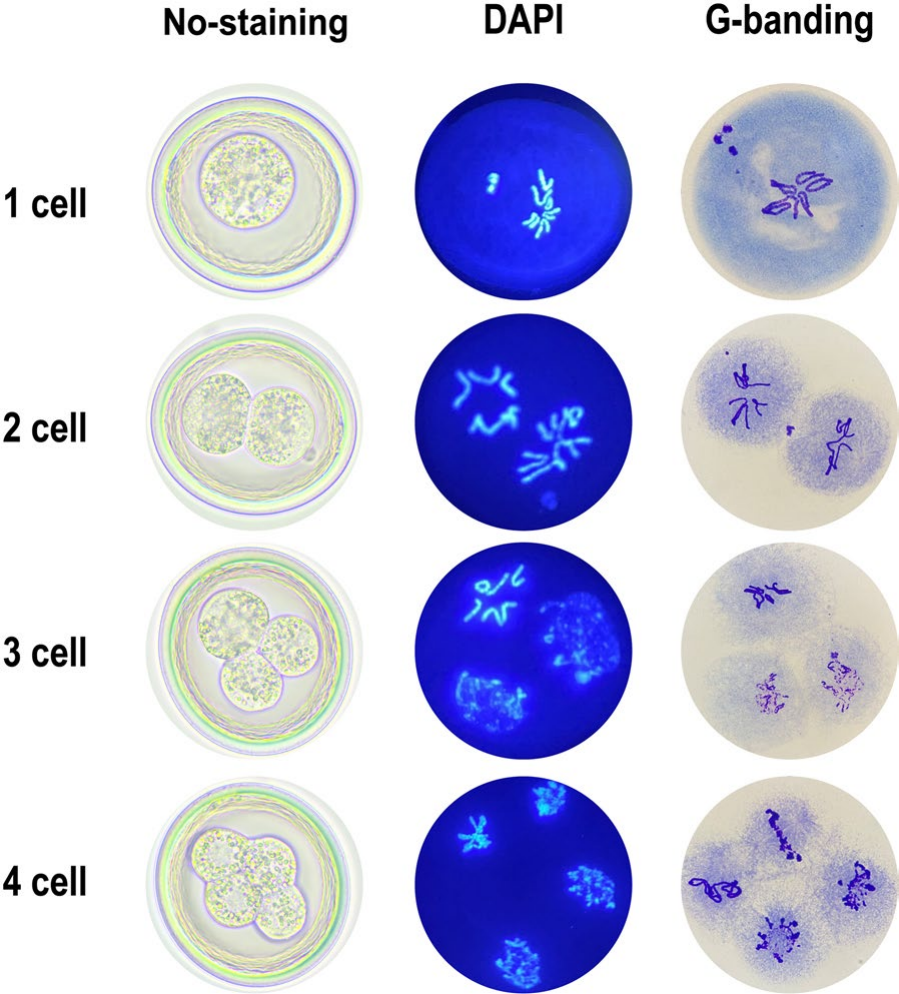
Karyotyping of early embryos at the 1–4-cell stages of the roundworms collected from donkeys in Liaocheng. From left to right: no staining, DAPI staining and Giemsa staining

Karyotyping was performed with eggs recovered from worms of three different hosts in China (in total 20 worms). Representative pictures of stained eggs are shown in Fig. 2. The roundworms collected from horses and zebras showed only two large chromosomes and were therefore assigned to the species *P. univalens*. In comparison, the roundworms (*Parascaris* sp.) collected from the donkey in Liaocheng showed six large chromosomes. The chromosome breakage occurred during the development of the eggs, and chromatin diminution occurred in pre-somatic cells of *Parascaris* sp. like *A. suum* and *P. univalens* (Fig. 3).

It was found that the spiculae of *P. univalens* terminate in a truncated and slightly concave form. In contrast, in *Parascaris* sp., they terminate in a distinctly rounded form (Fig. 2). Additionally, the chitinous layer of *Parascaris* sp. (> 5 μm) was thicker than that of the *P. univalens* (< 5 μm, *F*_(2537)_ = 1967, *P* < 0.01, Additional file 2: Table S1). Furthermore, the correlation coefficient between the size and the thickness of the chitinous layer of the eggs indicates no correlation (|*r*|< 0.3, *P* > 0.05, Additional file 1: Fig. S1). It is worth noting that the eggs in roundworms from the horses and donkeys were significantly larger than those from the zebras (*P* < 0.01).

**Fig. 4:**
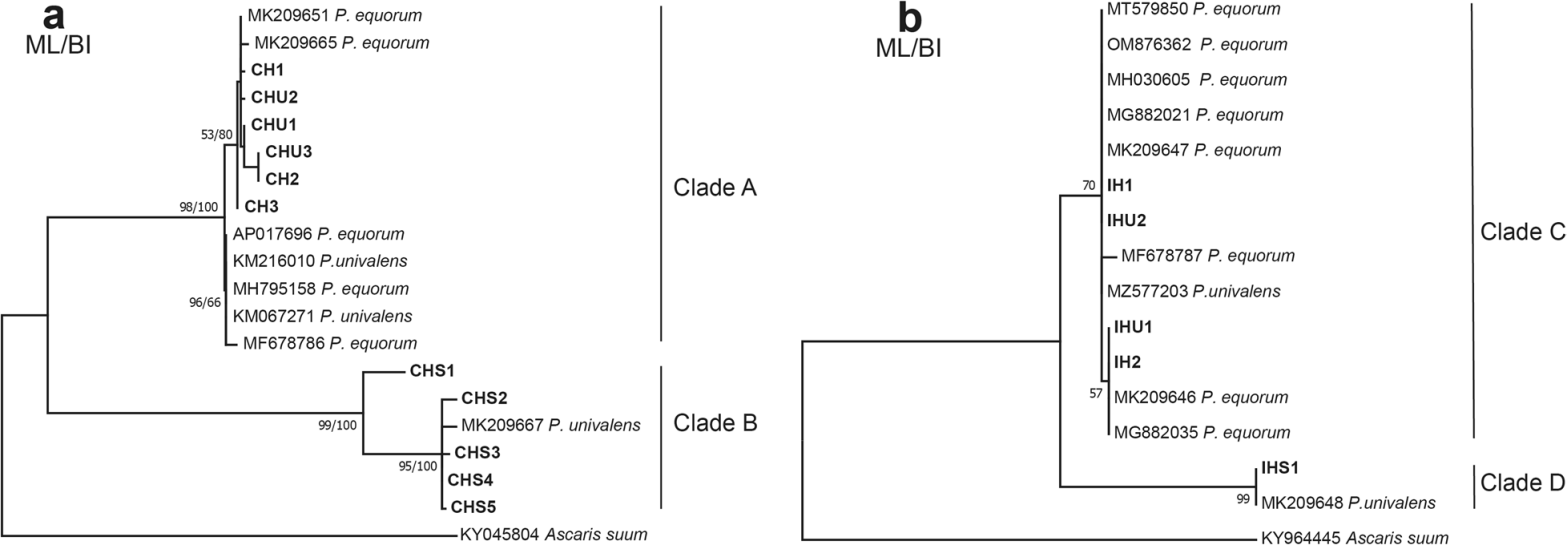
Phylogenetic relationship among *Parascaris* spp. based on the *COI* (**a**) and ITS (**b**) sequences using maximum likelihood (ML) and Bayesian inference (BI). Values higher than 50 are displayed on the trees. Bold indicates the sequence of this study. For *COI*, the sequences of *P. univalens* samples in horses and zebras were divided into three haplotypes (CHU1–CHU3); the *Parascaris* sp. in donkeys in Liaocheng were divided into five haplotypes (CHS1–CHS5); the sequences of samples in donkeys in Chifeng were divided into three haplotypes (CH1–CH3). For ITS, the sequences of *P. univalens* were divided into two haplotypes (IHU1 and IHU2); the *Parascaris* sp. were divided into one haplotype (IHS1); the sequences of samples in donkeys in Chifeng were divided into two haplotypes (IH1 and IH2)

The topology of the phylogenetic trees (Fig. 4) obtained from the ML analysis did not conflict with the BI trees. The *COI* gene tree showed that all five haplotype sequences (CHS1–CHS5) representing 14 worms from donkeys in Liaocheng formed a distinct clade (clade B). Sequences of the worms isolated from the horses, zebras and donkeys in Chifeng were randomly dispersed within clade A. Meanwhile, the ITS tree did not conflict with the *COI* trees, and all sequences were divided into two branches (clades C and D).

The roundworms of d1−d14 and d15−d24 were isolated from the donkeys, but they were split into two different clades (Additional file 3: Table S2, Fig. 4), which indicated that there may be more than one species of roundworm in donkeys (*P. univalens* and *Parascaris* sp.).

## Discussion

In previous work, we performed phylogenetic analysis without karyotyping of roundworms from horses, zebras and donkeys [5]. In this study, the karyotyping of eggs from different stages (1–4-cell stages) of *Parascaris* sp. showed that the pre-somatic cells underwent chromosome breaks, while the germline cell maintained intact chromosome morphology, consistent with the *P. univalens* studied by Müller et al. [22].

The only morphological identification study was performed more than 40 years ago by Biocca et al. [23], who found weak morphological differences in the terminal part of the spicula of the two species *P. univalens* and *P. equorum.* In this study, we also found differences in the terminal part of the spiculae in *P. univalens* (concave) and *Parascaris* sp. (rounded). Additionally, it was found that the egg’s chitinous layer of *Parascaris* sp. (> 5 μm) was significantly thicker than in *P. univalens* (< 5 μm) (*F*_(2537)_ = 1967, *P* < 0.01). The thickness of the chitinous layer and the size of the eggs were not correlated (|*r*|< 0.3, *P* > 0.05, Additional file 1: Fig. S1). Therefore, the thickness of the chitinous layer of eggs may serve as a diagnostic indicator to distinguish these two *Parascaris* species.

The phylograms showed the relations based on *COI* (Fig. 4a) and ITS (Fig. 4b), which revealed very close relationships between most of the sequences, regardless of whether they were deposited in GenBank as *P. univalens* or *P. equorum* in clades A and C. However, *Parascaris* sp. was a mono group in clades B and D. The phylogenetic analysis based on these sequences confirmed the karyotype identification results indicating that the worms from the donkeys in Liaocheng were not *P. univalens*. This study also suggests that some of nucleotide sequences deposited as *P. equorum* in GenBank were actually derived from *P. univalens* specimens as Samson-Himmelstjerna has stated [13].

The results of the haplotype information (Additional file 3: Table S2) and phylogenetic tree (Fig. 4) showed that donkeys could be infected not only by *Parascaris* sp. but also by another species, which may be *P. univalens*. Unfortunately, karyotyping of the suspected *P. univalens* in the donkey could not be carried out due to our poor preservation. Further study is necessary to investigate whether donkeys can be infected with different *Parascaris* species and whether they are reproductively isolated.

*Parascaris trivalens* with three pairs of chromosomes collected from horses was first described in 1934 [9]. The roundworm *Parascaris* sp. found in the donkey in the present study also has three pairs of chromosomes and could be recognized as *P. trivalens*. However, *P. trivalens* was found in horses nearly a century ago. There was no molecular biology information and only some hand-drawn figures of karyotypes without chromosome breaks [8, 9]. Therefore, the *Parascaris* sp. with six chromosomes in the donkey in the present study may be the species of *P. trivalens* described in 1934, but the possibility that it is a new *Parascaris* species cannot be ruled out.

Li posited that the *P. trivalens* he studied may be a six-chromosome polyploidy, the *P. equorum* is a tetraploid, and the *P. univalens* is amphiploid [9]. There were two possible rationales for this case. One was that higher polyploidy series were derived from lower ones by duplication; another way to explain the origin of the various types of *Parascaris* was by eliminating pairs of chromosomes, and the *P. trivalens* was the most primitive form [8, 9]. The *P. trivalens* has not been described in the literature since 1937, and karyotype identification of *P. equorum* with certainty in the horse has been absent since 1989 [24]. Meanwhile, *P. univalens* with two chromosomes have been recorded continuously [6]. If *P. trivalens* is a hexaploid and *P. equorum* is a tetraploid, why has *P. trivalens* not been recorded for nearly a century and why has *P. equorum* been absent since 1989 after it was karyotyped? If the *P. trivalens* and *P. equorum* were different *Parascaris* species and not the polyploidy of *P. univalens*, had they become extinct as endangered species in history? These phenomena deserve further exploration in the future.

## Conclusions

This study is the first report to describe a *Parascaris* species with six chromosomes in donkeys. It is worth noting that the thickness of the chitinous layer of the *Parascaris* egg may serve as a diagnostic indicator to distinguish the two ascarids (*P. univalens* and *Parascaris* sp.). The *Parascaris* sp. with six chromosomes in donkeys in the present study may be the species of *P. trivalens* described in 1934, but the possibility that it is a new *Parascaris* species cannot be ruled out.

## Supplementary Information

**Additional file 1: Figure S1.** Correlation analysis of the size and chitinous layer thickness of eggs in different populations of *Parascaris* spp. **a** Roundworms from horse. **b** Roundworms from zebra. **c** Roundworms from donkey.

**Additional file 2: Table S1.** The morphological information of samples in this study.

**Additional file 3: Table S2.** The molecular information of samples in this study and sequences in GenBank.

## Data Availability

The data that support the findings of this study are openly available in GenBank numbers OP745976, OP745979, OQ517636, OP745988, OP745989, OP745991, OQ517637 OP745984, OQ628067, OQ628069, OQ628068 (*COI*) and OP747659, OP747663, OP747671, OP747667, OP747668 (ITS).

## Abbreviations

DAPI: 4′,6-Diamidino-2-phenylindole
G-banding: Giemsa banding staining
