# Supplementary figures and images for: Exploration of Parascaris species in three different Equus populations in China
Source: Parasit Vectors. 2023 Jun 15;16:202. doi: 10.1186/s13071-023-05768-3 (PMC10273639; doi:10.1186/s13071-023-05768-3)

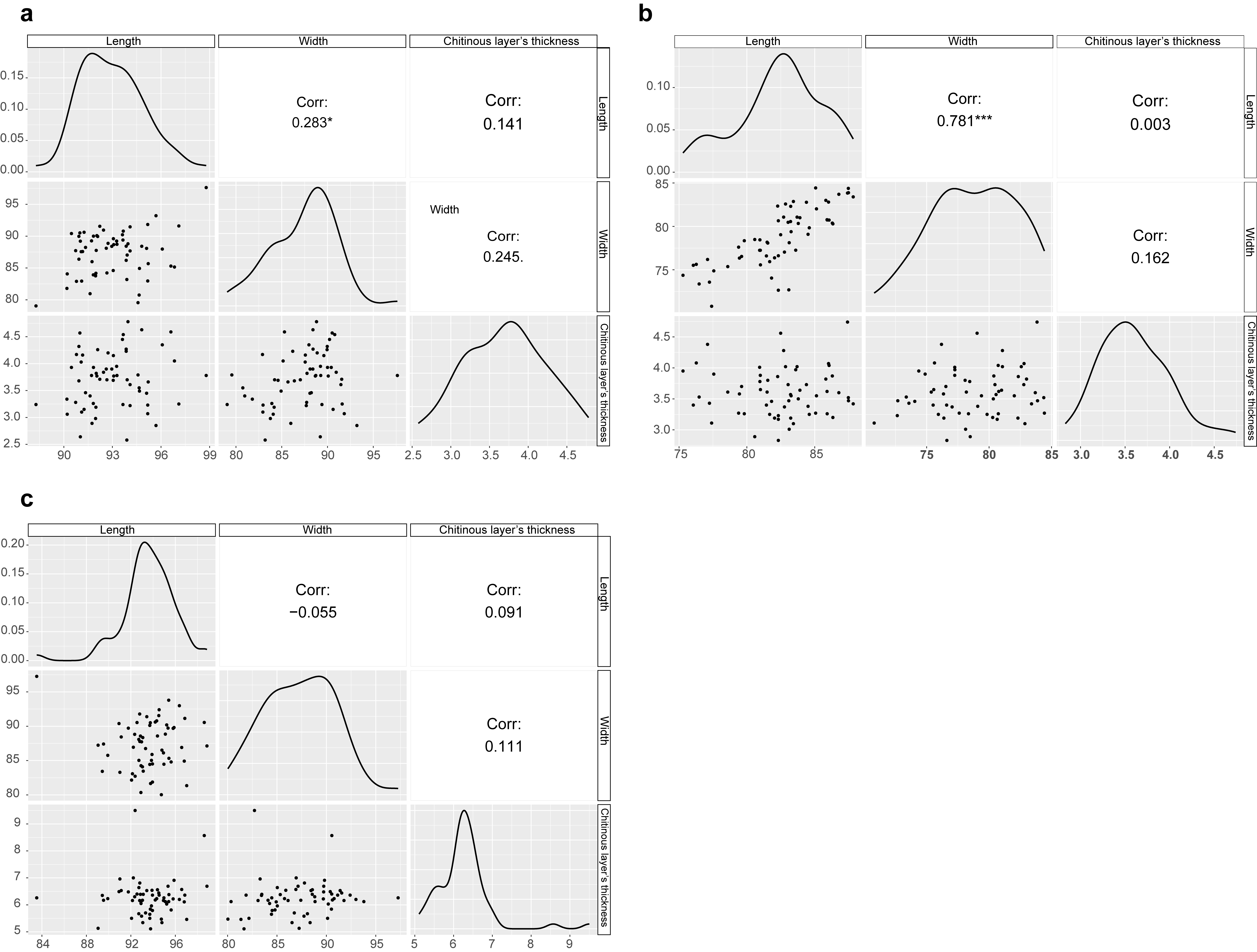

Supplement: Supplementary file 1 — Additional file 1: Figure S1. Correlation analysis of the size and chitinous layer thickness of eggs in different populations of Parascaris spp. a Roundworms from horse. b Roundworms from zebra. c Roundworms from donkey. [file 13071_2023_5768_MOESM1_ESM.tif]
